# Medical Items

**Published:** 1897-04

**Authors:** 


### MEDICAL ITEMS.

THE *Atlanta Clinic* is *non est.* *De mortuis nil.*

DR. E. C. DAVIS has returned from a pleasant visit to the schools and hospitals in New York and Philadelphia.

DR. A. H. VAN DYKE has returned from New York, where he spent several months in studying the eye, ear, nose, and throat. He will practice this specialty in the future.

DR. IRVING C. ROSSE, of Washington, D. C., does not believe in hydrophobia; and he backs his opinion with a standing offer of one hundred dollars to any one producing a genuine case in either dog or man.

AN appreciative subscriber writes: "Enclosed find $2.00 for which please continue my subscription to your journal. I can't do without it." This is the right sort of subscriber to have; and he knows a good thing when he sees it.

IF a subscriber notifies us to stop his JOURNAL and it is not done, or, if he orders his address changed and it is not, he may know that we did not get his order. Moving to another address without notifying us, does not excuse him from paying his bill.

AT a recent meeting of the Board of Trustees of the Jefferson Medical College, Philadelphia, Dr. J. Chalmers DaCosta was elected Clinical Professor of Surgery. Dr. DaCosta has been connected with the college for many years, and has recently been Demonstrator of Surgery and Chief of the Out-Patient Department.

THE New York legislature has enacted a law making it a felony for a person other than a duly licensed physician to have an anesthetic on his person with the intention of administering the same to another person, and makes the finding of the same on the person by any one presumptive evidence of guilt.

---

THE report of the special committee of the New York Academy of Medicine (Drs. Geo. R. Fowler, George Henry Fox, Henry J. Piffard, and A. A. Smith), to investigate the question of the contagiousness of leprosy, declares that leprosy is not contagious in this climate, and that it is unjust to segregate lepers, although steps should be taken to prevent the further landing of lepers.—*Virginia Medical Semi-Monthly.*

---

IT was in attendance on a young primipara. She suffered very much and chloroform was administered. Her mother, a very pious and sympathetic old lady, remarked to her: "My daughter, when I was in labor I always got a good deal of relief by repeating the twenty-third Psalm. Now repeat after me. 'The Lord is my shepherd, I shall not want.'" Daughter—"The Lord is my shepherd, I shall not wa'—wa'—wa'. I want some chloroform! Mamma! don't bother me now with psalms!"—*Atlantic Medical Weekly.*

---

DURING the year 1896 twenty-two so-called Keeley gold cures suspended and dissolved. Twenty-seven gold-cure homes, where specific treatment for alcohol and opium was given, have gone out of business. Five new companies have been formed to sell rights to use secret inebriate cures. Three ex-superintendents of gold-cure establishments have committed suicide. To this we would add that in three years we have made notes of the relapse of nineteen physicians who have been medical directors of gold-cure establishments. Ten of these persons came for treatment in regular asylums where no specifics were used.—*Journal of Inebriety.*

BEAR in mind, busy doctor, that the best contribution to a medical journal is often not the formal treatise, going back to first principles and giving everything up to the final completion of the subject, but the short note that can be made in a few minutes' interval between cases, stating some reliable new point in diagnosis, some valuable improvement in treatment, or a warning against some likely error. Also remember that the most of the contributions to current literature are made by the busiest men—those who, you would think, can scarcely find time for their meals. Kindly give the best results of your experience and observation, and in the briefest possible manner.—*Ex.*

---

THE next Annual Session of the American Medical Association will be held in Philadelphia June 1, 2, 3 and 4, commencing Tuesday at 10 A.M. It will be the semi-centennial session, and no pains will be spared to make it a particularly notable and successful meeting. A large attendance is expected and desired, and all who can do so should make it a point to be present. The President is Dr. Nicholas Senn, of Chicago. The address in Medicine will be delivered by Dr. Austin Flint, of New York; that in Surgery by Dr. W. W. Keen, of Philadelphia; and that in State Medicine by Dr. John B. Hamilton, of Chicago. The Chairman of the Committee of Arrangements is Dr. H. A. Hare, 222 South 15th Street, Philadelphia.

---

THE Congress of American Physicians and Surgeons will be held in the Columbian Theater, Washington, D. C., May 4th, 5th, and 6th. Special subjects for discussion will be "The Gouty and Rheumatic Diatheses, and their Relation to Diseases of the Eye"; "Otology in its Relations to General Medicine"; "Internal Secretions Considered in their Physiological, Pathological and Clinical Aspects"; "Deformities of the Hip-joint, especially Congenital Dislocations"; "Classification of Acute General Peritonitis, Prognosis and Treatment of Different Varieties." A large number of prominent American physicians and surgeons will be present and participate in the discussions. The President of the Congress is Dr. William H. Welch, of Baltimore; the Secretary is Dr. W. H. Carmalt, of New Haven, Connecticut.

---

THE NEW WOMAN.—The following is going the rounds:

" I can't conceive," she archly cried,
" Wherein you men can longer pride
Yourselves from female rivals free,
For surely we have grown to be
Your peers in every human stride.
It is a truth that none dare hide;
Yet why you men will not agree
To recognize the new degree,
I can't conceive.

" Now, *entre nous*, won't you confide,
And tell me true, all jokes aside,
What difference the world can see
Between your manly self and me?"
" To tell you truly," he replied,
"I can't conceive."

---

FRANCE to-day, with all her industry, natural resources and ardent national life, finds herself face to face with the specter of a decadent birth-rate, and her statesmen are planning, by the modification of the inheritance tax-law, to inspire a desire on the part of parents for more children. In brief, by exemptions to families containing more than three children and by additional taxes on those with less, they hope to counteract the alarmingly crescent tendency of French married couples to rest content with very few "olive branches." In 1894 the birth-rate in France was twenty-two to every 1,000 inhabitants—a decrease of 2.7 in a decade. This is believed to be the lowest birth-rate in the world, and no wonder thoughtful Frenchmen are aghast; and to this must be added the facts that illegitimate births and divorces are on the increase. Our condition is by no means so bad, but it is bad enough to demand prompt attention and remedial efforts, if Dr. Billings' latest figures be trustworthy. In 1880 he says our birth-rate was 30.95 to every 1,000 people, and in 1890 it had dropped to 26.68. If it has pursued to now the descending ratio, it must be, as we write, about 24.50—not three per cent. higher than the rate which frightens France. It appears further from the eminent sociologist's researches that the class of parents best equipped to bring forth and educate children are the least inclined to this patriotic responsibility.—*The New York Press.*

